# You have no power here! Social status does not modulate observationally acquired binding and retrieval effects

**DOI:** 10.1007/s00426-025-02191-4

**Published:** 2025-09-30

**Authors:** Kira Franke, Klaus Rothermund, Bernhard Hommel, Carina G. Giesen

**Affiliations:** 1 Department of Psychology, General Psychology II, Friedrich Schiller University Jena, Am Steiger 3, Haus 1, 07743 Jena, Germany; 2 Department of Psychology, Shandong Normal University, Jinan, Shandong Province China; 3 Department of Psychology, Faculty of Health, Health and Medical University, Erfurt, Germany

**Keywords:** Stimulus-response binding, Event files, Observational learning, Social status, Online interactions

## Abstract

Previous research shows that observationally acquired stimulus-response binding and retrieval (oSRBR) effects only occur when the observed person is socially relevant. An important factor that influences one’s social relevance in everyday life is social status: People typically orient their behavior and attention towards others whom they perceive to be of higher social status than themselves. This implies that these persons are considered more relevant than other persons of lower status. Thus, in two experiments we tested whether social status modulates oSRBR effects. To manipulate social status, participants were either assigned to a high status or low status condition. High status participants were able to give their interaction partner feedback on their performance and decided how an extra reward would be split between them. Then they performed an online interactive color classification task to assess oSRBR effects. Contrary to our expectation and despite successful manipulation checks, we did not find a modulation of oSRBR by social status in two highly powered (total *N* = 329) experiments. We discuss the implications of our findings for research on the influence of social status on imitative action regulation processes.

**Supplementary Information:**

The online version contains supplementary material available at 10.1007/s00426-025-02191-4.

## Introduction

According to the Theory of Event Coding (Hommel et al., 2001), executing a response in close temporal proximity to a stimulus, for example pressing a certain key in response to the color of a word presented on a screen, suffices to bind their mental representation together into a transient event file or stimulus response (SR) binding. If the same stimulus is encountered again, the SR binding is retrieved from memory, reactivating the response that was previously bound. If the retrieved response is also appropriate in the current situation, this leads to response facilitation, while it interferes with responding if the retrieved response is inappropriate (Rothermund et al., 2005; for overviews see Frings et al., 2020, 2024).

### Stimulus-response binding and retrieval by observation

Notably, the formation of such SR bindings is not limited to self-performed actions, but they can also be created and later on retrieved, if the response to a stimulus is only observed in another person (Giesen et al., 2014, 2017, 2018, 2021; Giesen & Rothermund, 2022). This was first demonstrated by Giesen et al. (2014). In their study, dyads of two participants performed a shared color categorization task (observational SR binding task). In the prime trial (trial n-1), one participant (prime actor) had to categorize the color of a word stimulus. The other participant (prime observer) saw the same word, but not the color, and had to observe the actor’s response. In the subsequent probe trial (trial n) participants switched roles: Now the former prime observer became the probe actor and had to categorize the color of the word stimulus. Stimulus relation, i.e., whether the word stimulus repeated or changed from prime to probe, and the compatibility between observed prime responses and to-be-performed probe responses (i.e., compatible vs. incompatible) were manipulated orthogonally. Interestingly, although the probe actor only observed the other’s responses in the prime trials, the probe actors’ performance reflected a pattern consistent with SR binding and retrieval effects: When observed prime responses and to-be-performed probe responses were compatible, probe responses were faster in stimulus repetition probe trials compared to stimulus change probe trials. However, when prime and probe responses were incompatible, performance was slower on stimulus repetition probes than on stimulus change probes. The presence of this interaction of stimulus relation and response compatibility implies that simply observing the other person’s response to the word stimulus in the prime trial led to the formation of an observational SR binding in the probe actor, which affected their performance once it was their turn to respond in the probe trial.

Crucially, in the study by Giesen et al. (2014) these observationally acquired stimulus-response binding and retrieval (oSRBR) effects occurred only when situational interdependence between participants was created by instructing them to either cooperate or compete for an extra reward. When they worked independently, however, meaning that obtaining the reward only depended on one’s individual performance, no oSRBR effects emerged. In a second study, Giesen et al.(2018) found that not only this situationally induced but also chronic interdependence modulated oSRBR effects: Participants only retrieved observed responses if their interaction partner was their romantic partner, but not if they performed the task together with a stranger. These findings suggest that people only rely on observed actions to guide their own performance if the observed person is socially relevant to them in some way. This idea is further supported by evidence from paradigms investigating related phenomena in a social context that also find effect modulations by the social relation between interaction partners. For instance, the spatial compatibility effect indicative of co-representation in the joint Simon task (Sebanz et al., 2003) becomes stronger both if interdependence between co-actors is situationally induced (Iani et al., 2011; Ruys & Aarts, 2010), and if their relationship is close and positive (Hommel et al., 2009; Quintard et al., 2020; Shafaei et al., 2020).

Interestingly, the modulating factors that determine whether an observed action will be used to guide one’s own response bear a close resemblance to phenomena known from *Social Learning Theory* (Bandura, 1986). Bandura (1986) argued that people often acquire their behaviors by observing others. However, one does not copy every observed behavior. Instead, whether an action will be copied depends on whether the model attracts the observer's attention and appears worthy of imitation (Bandura, 1986), for example by being socially relevant to the observer. According to Giesen (2024), the underlying cognitive mechanism of this might be a weighing process identical to intentional weighing (Memelink & Hommel, 2013): When the observed model is considered particularly relevant the stimulus- and response-related features will automatically be attended more compared to a less relevant model. Consequently, these features are weighted more and receive stronger activation. This would make it more likely for these features to be integrated into an observationally acquired SR binding and retrieved later on.

### Observationally acquired SR bindings in online settings

The studies by Giesen et al. (2014, 2018) were both conducted in laboratory settings where the participants interacted face-to-face. With the growing use of social media in recent years, however, social interactions are not limited to face-to-face settings any more, but increasingly take place online (Kepios, 2024). Therefore, it is of interest whether binding and retrieval by observation are affected by the same processes in both contexts. To study retrieval of observationally acquired SR bindings in virtual interactions, Giesen and Rothermund (2022) developed an online version of the observational SR binding task. In their study, they found first evidence that oSRBR effects are also prone to social modulations in online interactions, as retrieval effects were only present for participants who believed to be interacting with another human but absent if participants believed to be performing the task with a computer. However, it remains unclear whether, if participants believe their interaction partner to be another human, retrieval of observationally acquired SR episodes can further be modulated by social relevance modulations, as is the case for dyadic interactions (Giesen et al., 2014, 2018). The present experiments are the first ones to directly test this possibility, using the participants’ relative social status compared to their partner’s status to manipulate social relevance.

### Social status – definition and effects on behavior and cognition

Social status can be defined as an individual’s relative rank within a social hierarchy (Mattan et al., 2017). Social hierarchies emerge in any society or group of individuals. The structure provided by these hierarchies are beneficial for social life, as stable social hierarchies generally minimize conflicts (Berger et al., 1980), and facilitate cooperation and coordination within groups (Anderson & Willer, 2014).

As the bases of a social hierarchy can differ between societies or contexts, there are numerous ways for an individual to obtain social status. According to the Dominance-Prestige Model by Cheng and Tracy (2014) these can be subsumed under two fundamental, but distinct pathways to social status. The first pathway is dominance, which refers to differences in hierarchy that typically result from fear that dominant individuals instill in subordinate individuals through intimidation and coercion. This, in turn, leads subordinates to comply with a dominant person’s demands. Alternatively, social status can be acquired through prestige. In this case, the high status individual is valued and respected because of their skills, knowledge, and/or success and others willingly defer to them because of that (Cheng et al., 2014).

Social status has several important consequences for the individual within their group. First of all, it impacts on how one is evaluated by others: While high status individuals are usually perceived rather positively (Mattan et al., 2017), those with low status are more likely to be disliked (Cozzarelli et al., 2001) and perceived as less competent (Durante et al., 2017). Second, status affects behavior. For instance, Bandura et al. (1963) found that already at the age of 4 to 5 years, children imitate the behavior of an adult who controlled desirable resources like toys and food more frequently than the behavior of another adult in a subordinate and powerless role. Also, visual attention seems to be biased towards high status individuals. In fact, people are more likely to follow the gaze of a higher status than a low ranking individual (Dalmaso et al., 2012; Gobel et al., 2018) and tend to look more often and longer at high status than at low status individuals (Cheng et al., 2013; Foulsham et al., 2010). Powerful individuals, in turn, display a reduced tendency to adjust to others’ perspectives (Galinsky et al., 2006).

Additionally, there is some evidence from joint action research that suggests that social status also has an influence on automatic and implicit cognitive processes in situations in which two people work together on a task: In a study by Aquino et al. (2015), Italian participants performed the joint Simon task (Sebanz et al., 2003) either with another Italian or with an Albanian co-actor. The Albanians’ status was perceived as lower relative to the Italians’ social status, as the authors verified in a pilot study. Importantly, the spatial compatibility effect indicative of co-representation of the other’s task was only present when Italian actors worked with another Italian (i.e., someone with the same status), but not with an Albanian (i.e., someone with comparably lower status) co-actor, which suggests that relative social status can influence co-representation (Aquino et al., 2015). Relatedly, van der Weiden et al. (2021) found a modulation of the joint Simon effect by participant’s seating position (elevated vs. low), which impacts feelings of power. Further, Tufft (2022) found that social offloading processes (i.e., ignoring distracting information that is deemed the responsibility of the interaction partner) are sensitive to the interaction partner’s perceived status manipulated via visual cues and CV information. Participants socially offloaded only when they interacted with a partner of high status but not with a partner of relatively lower status. Also, there are two studies that found evidence that automatic imitation effects were stronger for participants with a lower social status compared to those with a higher status (Farwaha & Obhi, 2021a, b).

### Research aim and hypothesis

In sum, previous research shows that social status has a strong influence on people’s perception and attention towards other individuals and can even affect the degree to which observed actions are co-represented or utilized for one’s own action regulation. Tentatively, this suggests that social status also plays an important role in how socially relevant someone is perceived by others. Consequently, in line with previous research (Giesen et al., 2014, 2018), social status might also modulate retrieval of observationally acquired SR bindings. However, to date, this has never directly been tested. Therefore, in the present study, we investigated whether social status modulates oSRBR effects. Additionally, this allowed us to get further insight on how sensitive retrieval of observationally acquired SR bindings is to modulations by social relevance in online interactions.

To manipulate social status, participants were randomly assigned either the role of *leader* in the high status condition or the role of *follower* in the low status condition before performing the online version of the observational SR binding task. Leaders’ social status was elevated compared to their interaction partner’s by having them evaluate their partner’s performance at several times during the experiment. Furthermore, leaders had the power to decide how an extra reward would be split between both interaction partners at the end of the experiment. In contrast, follower’s performance was evaluated after each block and they had to wait for their interaction partner’s decision in all of these situations. As a result, participants assigned to the role of follower represent the low status condition and should regard their higher status partner as particularly socially relevant. This should in turn increase the attention they pay to the responses executed by their higher status partner, benefiting both encoding and retrieval of SR episodes (Logan, 1988; Moeller & Frings, 2014). Participants with the role of leader represent the high status condition and should perceive their partner as less relevant and therefore attend less to their responses, hindering the emergence of oSRBR effects. Statistically, this would be reflected in a significant three-way interaction of stimulus relation, response compatibility and social status. Specifically, we expected the stimulus relation x response compatibility interaction to be significant in the low status condition, indicating oSRBR effects. In the high status condition, oSRBR effects should be significantly reduced or absent, which means that there should be no interaction of stimulus relation and response compatibility. To anticipate results, in Experiment 1 we found significant SR retrieval effects in both conditions, independently of social status. However, the observational SR binding task in Experiment 1 included an occasional memory test, in which participants were asked to remember the last response they had observed their partner perform. To control for the possibility that this test alone increased attention sufficiently for oSRBR effects to occur, masking potential modulating effects of the social status manipulation, we conducted Experiment 2 as an almost exact replication of Experiment 1, except that the memory test was removed.

## Method

### Preregistration, open access and ethics vote

Experiment 1 was preregistered online at the Open Science Framework (OSF) prior to data collection (https://osf.io/wegvh). For both experiments, experimental files, data, and analyses scripts will be made available (https://osf.io/d6wq2/).

All experiments were in accordance with the Ethical standards of the Institute of Psychology of the FSU Jena and the Declaration of Helsinki. For both experiments, all participants gave informed consent via key press prior to taking part.

### Participants

We ran an a priori power analysis in G*Power 3.1 (Faul et al., 2007) to estimate the required sample size. In previous online studies, Giesen and Rothermund (2022) obtained an effect size of *d*_*z*_=0.39 for modulations of oSRBR effects. To detect an effect of this size with a statistical power of 1-β = 0.80 and an alpha level of α = 0.05 in a one-tailed independent-samples *t*-test, a total of *n* = 164 (82 per status condition) participants are needed.

For Experiment 1, a total of *n* = 203 participants was recruited online from Prolific Academic (https://www.prolific.com/). They were prescreened to be Native English speakers, currently located in the UK, aged between 18 and 45 years, conducting the experiment on a computer or notebook and with no previous participation in online experiments on oSRBR effects. In line with the preregistration, 36 participants had to be excluded due to excessive error rates (> 25% errors in the memory test), one participant was excluded because of missing data. Thus, data of *n* = 166 participants were analyzed (83 female, 83 male, *M*_*age*_ = 33.1 years, *SD*_*age*_ = 6.7).

For Experiment 2, *n* = 164 new participants were sampled from Prolific Academic with the same pre-screen criteria. One person was excluded because of incomplete data, resulting in the data of *n* = 163 participants being analyzed (102 female, 60 male, 1 diverse, *M*_*age*_ = 33.9 years, *SD*_*age*_ = 6.7).

The medium duration of Experiment 1 was 27 min; for Experiment 2 it was 24 min. After each experiment, all participants received a financial reward of £4.50 (5.29€) for taking part. Additionally, they could obtain an extra reward between £0.00 and £0.42.

### Apparatus and stimuli

Both experiments were programmed and hosted online using the Gorilla Experiment Builder (www.gorilla.sc; Anwyl-Irvine et al., 2020). Participants conducted the studies on a desktop computer or notebook. We used 25 neutral, monosyllabic or disyllabic English adjectives (e.g., warm, slow, even) as stimuli. Stimuli were presented centrally on a black screen in 30px font size.

### Procedure

Both experiments followed the same procedure unless mentioned otherwise. At the beginning of each experiment, demographic information was collected. Next, participants were informed that they would perform an interactive response time task with another person and that they would be digitally connected with this person now. Participants waited a maximum of 5 min to be connected, if no other person was found, the study was terminated and participants received a partial compensation of £0.75. In case of a successful match, the participant pairs then had three minutes to chat with each other via text messages. As in a previous study, oSRBR effects were only present for participants who believed to be interacting with another human (Giesen & Rothermund, 2022), this was done to convince participants that they were doing the experiment together with another human. However, after the chat, participants were disconnected from each other without their knowledge and interacted with a computer program for the rest of the experiment. To maintain the illusion of a real interaction during the experiment, they sometimes had to wait for their interaction partner to finish reading instructions or executing responses.

Participants were then randomly assigned to either the high status (Experiment 1: *n* = 82; Experiment 2: *n* = 82) or the low status (Experiment 1: *n* = 84; Experiment 2: *n* = 81) condition. In the high status condition, participants were assigned the role of *leader*, whereas participants in the low status condition were assigned the role of *follower*. They were told that the other participant had been assigned the other role, and that they would from now on always be referred to as the leader or the follower, respectively. Furthermore, it was explained that, over the course of the experiment, they would each gain points for a shared extra reward, which would be paid out as additional monetary compensation (£0.01 for 1 point), based on their performance. Leaders received information about the follower’s performance and the points gained by the follower several times during the experiment and were asked to evaluate them after that. They also decided how the extra reward would be split between them at the end of the experiment. Followers, on the other hand, simply had to wait for the leader’s decision in both scenarios. They therefore also did not know who had gained how many points for the shared reward.

**Fig. 1:**
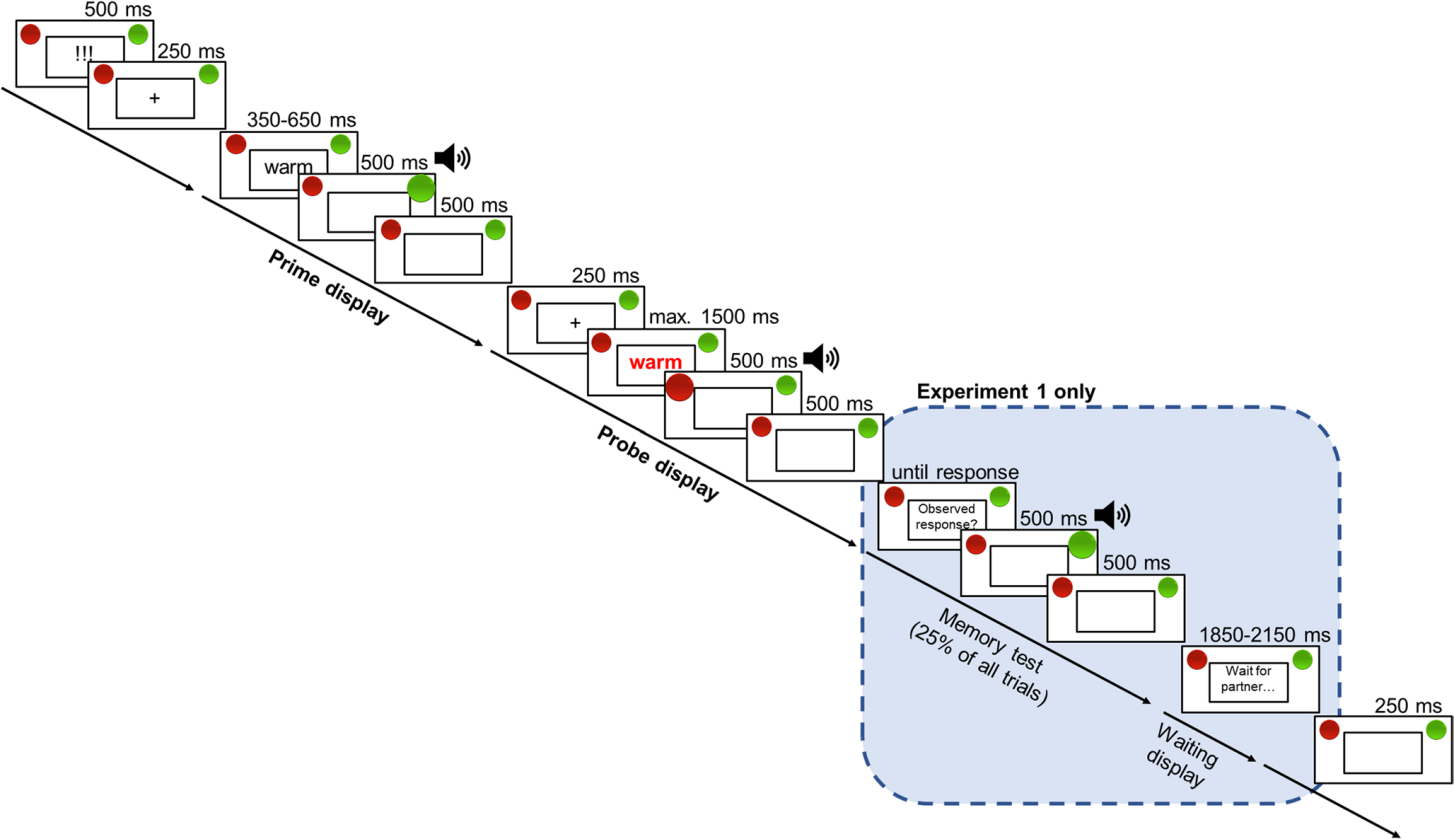
Example of a prime-probe sequence in the observational SR binding task. Note. Stimuli are not drawn to scale. For illustrative purposes, foreground and background colors are inverted. Stimuli in boldface were presented in red/green; stimuli in normal face were presented in white. In Experiment 2, the memory test (highlighted) was removed

To assess oSRBR effects, participants performed the online version of the observational SR binding task developed by Giesen and Rothermund (2022). Participants were informed that in this task, words would appear centrally on their screen in a squared rectangle, and that they had to categorize these words based on the color. The task was taken in turns. If participants saw a word in red or green font, they had to respond as fast and accurately as possible, by pressing ‘A’ (left key) for red and ‘L’ (right key) for green. After a response had been made, a virtual red or green response button lit up in the upper right or left corner of the screen, accompanied by a clicking sound (see Fig. 1). However, if a word appeared in white font, it was the other person’s turn to respond. In this case, participants were instructed to observe their partner’s response, which was represented in the same way as their own responses, by virtual red or green response button lighting up and a clicking sound. In Experiment 1, they were also instructed to memorize the partner’s response, as they would be asked to remember it in occasional memory tests.

After reading all instructions, participants had to complete a brief instruction check consisting of two questions. If they did not answer both questions correctly, they had to read all instructions a second time. Before the start of the task, they were asked to turn on their loudspeakers or wear headphones and to place their index fingers on the ‘A’ and the ‘L’ keys.

Before the start of the experiment’s main block, participants completed a practice block of 16 prime-probe sequences. They received error feedback for incorrect categorizations (“WRONG KEY!”), slow responses (“Respond faster!”), responding out of turn (“WRONG PERSON!”), and, in Experiment 1 only, for incorrect responses in the memory test (“INCORRECT!”). The practice block was repeated if participants made more than 25% errors in the color classification task, responded too slow (> 1500ms) in more than 25% of the prime-probe sequences, or responded when it was not their turn more than two times, or in Experiment 1, if they made more than 25% errors in the memory test. If participants did not pass the practice block on their second try, the experiment was terminated.

In the main block of the experiment, participants worked through 128 prime-probe sequences (see Fig. 1). Each prime-probe sequence started with a ready signal (“!!!”, 500ms) presented centrally on the screen in white font on a black background. Then the prime trial started with a fixation cross (250ms), followed by the appearance of a white word, which remained on the screen between 350 and 650 ms. Once the word disappeared, either the red or the green response button lit up in one of the upper corners of the screen. This illusion was created by showing a larger picture of one of the buttons for 500ms and then showing it in its standard size for another 500ms. Simultaneously to the presentation of the larger button, a clicking sound was played. If the participants had responded to the white word, error feedback was shown (“WRONG PERSON!”, 1000ms). After that, the probe trial began with another fixation cross (250ms). Then a word appeared in either red or green font. The word remained centrally on the screen either until a response was executed by the participant by pressing either the ‘A’ or the ‘L’ key or until 1500ms had passed. Once a response had been made the word disappeared and the corresponding button lit up the same way as described for the prime trial. The sequence of white and colored trials created the impression that the participant and their partner were working on the trials of the task in strict alternation. Error feedback was shown for 1000ms, if an incorrect response had been given (“WRONG KEY”) or if the participant had not responded in time (“Respond faster!”). Each sequence ended with a blank screen with a duration of 250ms.

In Experiment 1, in 25% of the prime-probe sequence a memory test was presented after the probe trial. In the memory test, participants were asked to press the key that corresponded to the response they had previously observed in the prime trial. This request remained on the screen until the participant responded. Then the response button corresponding to the given response lit up, as described above. If the response was incorrect, error feedback was shown (“INCORRECT!”, 1000ms). In another 25% of prime-probe sequences, it was suggested that their partner was doing the memory test. In this case the sentence “Waiting for the follower/leader to respond…” was shown on the screen for a variable duration between 1850 and 2150ms.

After each block of 32 prime-probe sequences there was a break. During this break participants in the high status condition received scripted feedback about their partner’s (the follower’s) performance (% errors in the color categorization task, slow responses, and in Experiment 1 errors in the memory test) and they were informed how many points the follower had gained for their shared extra reward based on this performance. Leaders did not receive feedback on their own performance. They were also reminded that they would decide how the extra reward would be split between them and the follower at the end of the experiment. The information provided remained on the screen until the participant pressed the space bar. Then they were asked to rate the follower’s performance by clicking on a picture of a thumbs up or a thumbs down button. In the low status condition, no performance feedback was shown, participants were only reminded that they were being evaluated by the leader. Once they indicated that they were ready to continue with the task by pressing the space bar, a picture of either a thumbs up or a thumbs down was shown, suggesting that that was the feedback that the leader had chosen for them. However, which feedback was shown was determined randomly, with a 50% chance for each outcome.

After the observational SR binding task was completed, participants were informed that they had accumulated 42 points for the shared extra reward during the task together with their partner. This amount was always the same independently of their actual performance. In the high status condition, participants were asked to choose the ratio (follower: leader), in which the extra reward should be distributed. They were presented with 11 options ranging from 0:100 to 100:0, increasing in steps of 10 and confirmed their decision by clicking on the option. In the low status condition, participants had to wait for 7000ms and were then informed about the leader’s decision. It was preprogrammed that the reward would always be split with a 50:50 ratio in this condition.

In the final part of the experiments, participants were asked to write down what they thought the study was about and they were asked to remember which role they had during the experiment (options: follower, leader). Then, as a manipulation check they were asked a couple of questions about their own and their partner’s experience during the experiment. First, they were asked to rate via mouse click on a 9-point Likert scale how active, superior, independent, powerful and decisive they felt during the experiment (1 = very passive/inferior/dependent/powerless/indecisive, 5 = neutral, 9 = very active/superior/independent/powerful/decisive). Second, they were asked to rate the same items again from their partner’s perspective, meaning how they think their partner felt during the experiment. Last, participants were asked to indicate with whom they believed they had interacted while they were doing the experiment (options: another person vs. another person, but now I doubt this vs. never believed to be interacting with another person). After answering all questions, participants were fully debriefed.

### Design

 The experiments used a 2x2x2 mixed-factors design with the two within-subject factors stimulus relation and response compatibility and one between-subject factor, which was status. Stimulus relation was manipulated by either repeating the same word stimulus from prime to probe in 50% of all prime-probe sequences (stimulus repetition, e.g., slow‑slow) or changing the word stimulus presented from prime to probe in 50% of the prime-probe sequences (stimulus change, e.g. slow-warm). Response compatibility was manipulated by requiring participants to execute a probe response that was compatible with the observed prime response in 50% of the prime-probe sequences (e.g., red-red) or a probe response that was incompatible with the observed prime response in 50% of all prime-probe sequences (e.g., red-green). The between factor status was varied by randomly assigning participants to either the high status or the low status condition at the beginning of the experiment. Probe reaction times (RT) served as the dependent variable of interest.

### Data Preparation

Prior to analyses, we excluded all probe responses with erroneous responses in the color classification task (Experiment 1: 1.6%; Experiment 2: 2.2%) or in the memory test (Experiment 1: 7.1%; overall: 1.7%). Further, we discarded probe responses, if the participants had accidently responded to the prime stimulus in that prime-probe sequence (Experiment 1: <0.1%; Experiment 2: <0.1%). Also, probe responses faster than 200ms or slower than 1.5 interquartile ranges above the 75th percentile of the individual RT distribution were regarded as outliers (Tukey, 1977) and were excluded (Experiment 1: 3.5%; Experiment 2: 3.3%).

For each experiment, we computed mean probe RTs for each condition of the factorial design (see Table 1). Additionally, we computed effect scores for oSRBR effects representing the stimulus relation x response compatibility interaction for each participant (see Table 1 for computation).

**Table 1:** Probe performance M (SD) in the observational SR binding paradigm

		High status		Low status	
		C	IC	C	IC
Experiment 1	Stimulus repetition (SR)	506 (73)	506 (69)	498 (77)	502 (74)
	Stimulus change (SC)	515 (75)	503 (70)	506 (80)	500 (71)
	Δ SC - SR	9** [2.8]	−3 [1.9]	8*** [2.2]	−2 [2.3]
	S x R interaction score	12** [3.3]		10** [3.3]	
Experiment 2	Stimulus repetition (SR)	491 (70)	486 (71)	477 (54)	471 (57)
	Stimulus change (SC)	493 (70)	488 (74)	480 (58)	471 (56)
	Δ SC - SR	2 [2.1]	2 [2.0]	3 [1.7]	0 [2.1]
	S x R interaction score	0 [2.9]		3 [2.5]	

*Note. *C = compatible probe response, IC= incompatible probe response. S x R interaction score = (Δ SC - SR)_C_ - (Δ SC - SR)_IC_. Standard error of the mean in brackets

* *p* <.05. ** *p* <.01. *** *p* <.001. Asterisks denote that effects significantly differ from zero

## Results

Analyses were conducted in R (Version 4.1.2). Bayes Factor computations were performed with JASP (Version 0.14.1.0).

### Manipulation checks

#### Belief that the partner was human

Most participants (Experiment 1: 82%, Experiment 2: 69%) indicated that they either believed to have interacted with another human, or that they had believed it while doing the experiment and only doubted afterwards, while only a minority (Experiment 1: 18%, Experiment 2: 31%) claimed they had never believed their interaction partner was real. Percentages did not differ significantly between status groups neither in Experiment 1 (*t*(159) = 1.55, *p* =.124, *d* = 0.24, high status: 87%, low status: 77%), nor in Experiment 2 (*t*(161) = 0.29, *p* =.775, *d* = 0.04, high status: 68%, low status: 70%). This excludes the possibility of differences in retrieval effects being due to more participants doubting whether their partner was real in one group than in the other.

#### Status manipulation

To get a single score for how participants perceived their own status during the experiment compared to their partner’s status, we first calculated the mean rating across all items regarding how the participant felt during the experiment (*M*_*self*_) for each participant and the mean rating across all items on their partner’s feelings (*M*_*other*_*)*. Then we subtracted *M*_*other*_ from *M*_*self*_. Positive values on this status score indicate that participants perceived themselves higher in status compared to their partner; negative values indicate that they felt that their status was lower than their partner’s. Importantly, in both experiments scores differed significantly between status groups (Experiment 1: *t*(164) = 12.3, *p* <.001, *d* = 1.90, Experiment 2: *t*(158) = 12.1, *p* <.001, *d* = 1.90), with participants in the high status group perceiving themselves as higher in status (Experiment 1: *M* = 1.44, Experiment 2: *M* = 1.40) than participants in the low status condition (Experiment 1: *M* = −1.49, Experiment 2: *M* = −1.55). We can therefore conclude that the status manipulation was successful.

#### Memory test performance

For Experiment 1, participants’ average error rates in the memory tests were compared as a function of status group to ensure that potential differences in retrieval of observationally acquired SR episodes between status groups were not due to differences in their motivation to observe their partner’s responses. Error rates did not differ significantly between groups, *t*(164) = 0.94, *p* =.346, *d* = 0.15, implying that participants in both groups adequately attended to and memorized the prime responses in Experiment 1 (high status: *M* = 6.6%, low status: *M* = 7.6%).

### Probe performance

**Fig. 2:**
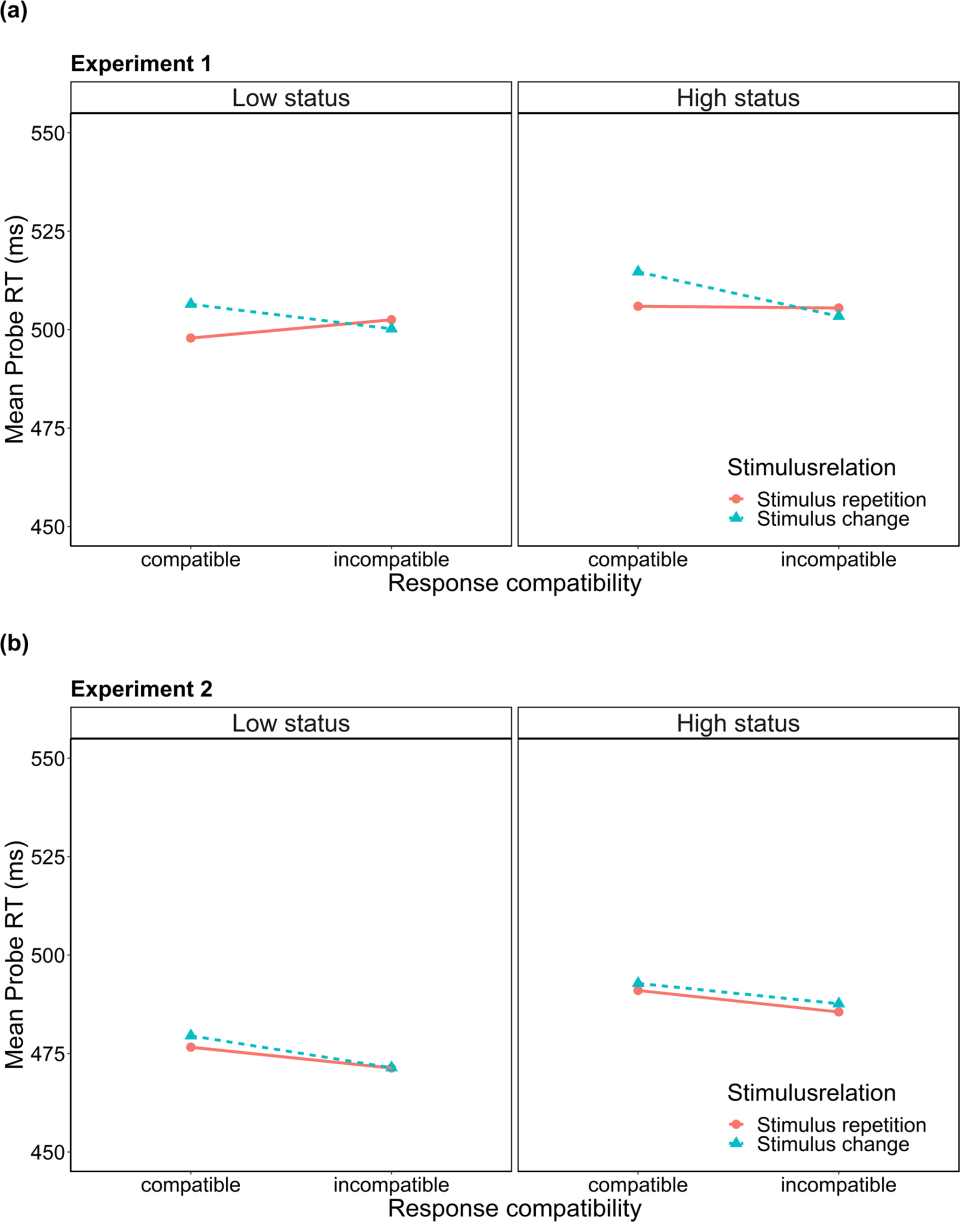
Probe performance (RT) as a function of stimulus relation, response compatibility, and status condition (**a**) in Experiment 1 and (**b**) in Experiment 2

To analyze probe performance, we conducted a 2 (stimulus relation: stimulus repetition vs. stimulus change) x 2 (response compatibility: compatible vs. incompatible) x 2 (status: high status vs. low status) mixed factors analysis of variance (ANOVA) on mean probe RTs. Results for Experiment 1 revealed a significant main effect of stimulus relation, *F*(1, 164) = 7.79, *p* =.006, *η*^2^_p_ = 0.05, with faster responses when the word stimulus repeated from prime to probe (*M* = 503 ms) compared to when it changed (*M* = 506 ms). There was also a significant interaction of stimulus relation and response compatibility, *F*(1, 164) = 21.9, *p* <.001, *η*^2^_p_ = 0.12, indicating the presence of oSRBR effects. Importantly and contrary to our prediction, the three-way interaction was not significant, *F*(1, 164) < 0.001, *p* =.988, *η*^2^_p_ < 0.01, implying that retrieval effects (i.e. the stimulus relation x response compatibility interaction) did not differ between status groups (see Fig. 2). All other effects did not reach significance (all *F* ≤ 3.33, all *p* ≥.07*)*.

For Experiment 2 the ANOVA revealed a significant main effect of response compatibility, *F*(1, 161) = 23.1, *p* <.001, *η*^2^_p_ = 0.13, indicating that responses were faster if the probe response differed from the response observed in the prime (*M* = 479 ms) than when responses were compatible (*M* = 485 ms). All other effects were not significant (all *F* ≤ 2.75, all *p* ≥.100). Crucially, both the two-way interaction of stimulus relation and response compatibility (*F*(1, 161) = 0.44, *p* =.508, *η*^2^_p_ < 0.01) and the three-way interaction(*F*(1, 161) = 0.64, *p* =.424, *η*^2^_p_ < 0.01) failed to reach significance. This implies that there was no retrieval of observationally acquired SR bindings in Experiment 2, and consequently and contrary to our hypothesis, retrieval effects also did not differ between status groups (see Fig. 2).

For a direct test of our directional hypothesis, effects scores for retrieval of observationally acquired SR bindings (see Table 1 for computation) were compared between status groups using a one-tailed, independent-sample *t*-test. The *t*-test revealed no significant difference, neither in Experiment 1 (*t*(164) = 0.02, *p* =.493, *d* = 0.00, BF_01_ = 5.88), nor in Experiment 2 (*t*(161) = 0.82, *p* =.212, *d* = 0.13, BF_01_ = 2.82). This indicates that effects scores were not significantly larger in the low status condition (Experiment 1: *M*_*SxR*_ = 10.9 ms, Experiment 2: *M* = 2.81 ms) than in the high status condition (Experiment 1: *M*_*SxR*_ = 10.9 ms, Experiment 2: *M* = −0.26 ms). Additionally, for each status group a two-tailed *t*-test against zero was conducted. The *t*-test was significant both in the high status, *t*(81) = 3.30, *p* =.001, *d* = 0.36, BF_01_ = 0.06, and the low status group, *t*(83) = 3.32, *p* =.001, *d* = 0.36, BF_01_ = 0.05, in Experiment 1. In Experiment 2, however, average effect scores did not differ significantly from zero in neither the high (*t*(81) = 0.09, *p* =.929, *d* = 0.01, BF_01_ = 8.18), nor the low status condition (*t*(80) = 1.11, *p* =.269, *d* = 0.12, BF_01_ = 4.50). These results imply that, contrary to our expectation, in Experiment 1 retrieval of observationally acquired SR bindings did not only occur in the low status group, but also in the high status condition, while in Experiment 2 there was no retrieval of observationally acquired SR bindings in any of the groups.

## Discussion

In two experiments we investigated the influence of social status on retrieval of observationally acquired SR bindings. We expected retrieval effects to only be present for participants interacting with someone whose status was higher than their own. For both experiments, the manipulation check clearly shows that social status was manipulated successfully: Participants in the high status condition indicated that they felt more powerful than their interaction partner, while participants in the low status condition felt less powerful than their partner. However, contrary to our predictions, SR retrieval effects did not differ as a function of social status^1^In Experiment 1, we found retrieval of observationally acquired SR bindings in both conditions, without any evidence of a modulation of oSRBR effects by social status. In Experiment 2, the memory test was removed because we speculated whether this counteracted the effect of the status manipulation. However, in Experiment 2, there were no retrieval effects in neither of the groups, despite successful manipulation checks. Even when combining the data of both experiments in a joint analysis (see supplementary material) no influence of social status on oSRBR effects could be detected.

There are several reasons that might account for why SR retrieval effects were not affected by social status. First, comparing the results of the two experiments, what stands out is that we only found a statistically significant interaction of stimulus relation and response compatibility indicative of SR retrieval effects in the first experiment. In contrast, this interaction was absent in the second experiment. The only difference between both experiments was that Experiment 2 did not include a memory test for observed prime responses. On the one hand, this indicates that the memory test is crucial to ensure that participants pay sufficient attention to observed responses. On the other hand, one could therefore argue that the knowledge that their memory would be tested alone increased participants’ attention sufficiently for the emergence of oSRBR effects, without the need for further social relevance factors like social status. However, considering the results of previous studies on observationally acquired SR bindings, this explanation seems unlikely. Several studies that had also included a memory test like in Experiment 1 found a modulation of oSRBR effects by different social relevance manipulations (Giesen et al., 2014, 2018; Giesen & Rothermund, 2022). Importantly, in all of these studies, there was no retrieval effect in the respective low social relevance condition (i.e., when participants worked independently, with an unrelated stranger, Giesen et al., 2014, 2018), in spite of the participants being tested for their memory for observed responses. Thus, the memory test by itself is necessary, but probably not sufficient for retrieval of observationally acquired SR bindings to occur. Rather, its function seems to be to maintain participants’ focus on the responses of the interaction partner to ensure their attention across the entire task. This is particularly important given the differences between the observational SR binding task and other non-interactive paradigms investigating binding and retrieval. Usually, in non-interactive tasks, the participants’ attention has to always be focused on the screen, since they have to respond in every trial. In the observational SR binding task this would not be necessary without the memory test: the partner’s responses do not affect how the participant has to respond when it’s their turn, which may lead them to not attend to the screen at all during prime trials. Attending to the prime trial’s stimulus and the emitted response however is crucial for the emergence of bindings between the stimulus and the observed response. This is supported by an exploratory analysis comparing the first vs. the second half of Experiment 2 (see supplementary material): Descriptively, there was a pattern reflecting retrieval effects in each status condition for the first half, but not for the second half. This might suggest that participants attended their partner’s responses as instructed at the start of the experiment but stopped paying attention (possibly due to fatigue/loss of concentration, etc.) later on.^2^

Another reason for the social status manipulation failing to modulate oSRBR effects could be that we unintentionally created interdependence between participants and their partner with the status manipulation we used. Participants were told that they could gain a shared extra reward based on both their performances, which might have been interpreted as working together towards a common goal. Thus, high status participants were not completely independent from their partner, since the amount of extra money they would receive depended partly on their partner’s performance. Interdependence has been demonstrated to be an effective way to create sufficient social relevance for retrieval of observationally acquired SR bindings to occur (Giesen et al., 2014; Giesen et al., 2018). Thus, this might have compensated for their partner’s lower status. Additionally, for leaders, the instruction to evaluate their partner’s performance might also have led them to pay more attention to their partners’ responses, which effectively counteracted the intended effect of the status manipulation.

Alternatively, the results could be explained by the nature of the experimental task itself. Participants might perceive working with their interaction partner on the color categorization task alone as cooperative enough for binding and retrieval by observation to occur automatically, unless the partner is regarded as particularly irrelevant. This idea is supported by the results of Giesen and Rothermund (2022), who found oSRBR effects in a condition where participants simply believed to be interacting with another human without increasing their social relevance in any other way. In all previous experiments that did show a modulation of oSRBR effects in interactions between humans, in the condition in which effects were absent, participants were explicitly instructed to work independently from their partner (Giesen et al., 2014, 2018), which is not the case for any of the conditions in the present experiments. Relatedly, the compatibility effects indicative of co-representation of another person’s actions in the joint Simon task usually occur without any additional induction of social relevance (Ferraro et al., 2011; Sebanz et al., 2003), and are only reduced or absent when additional factors are present that would create distance between the co-actors, like a negative relationship (Hommel et al., 2009) or a non-human co-actor (Müller et al., 2011; Tsai & Brass, 2007; Tsai et al., 2008). The present results, therefore, suggest that a partner’s low status did not lead to participants disregarding them as irrelevant enough for preventing oSRBR effects. This is differs from what is typically found in related paradigms like the joint Simon paradigm, where interacting with a lower status co-actor eliminated the joint Simon effect (Aquino et al., 2015). Also, Tufft (2022) found that participants did not socially offload to interaction partners with a lower social status. However, these findings can be reconciled with ours by taking a closer look at the specific social status manipulations employed in the experiments: In the study by Aquino et al. (2015), status was determined by nationality and Tufft (2022) manipulated status both through visual cues, specifically the looks of the co-actor’s clothes and apartment, and CV information. Thus, both studies, in contrast to ours, used (seemingly) authentic and personal information about the individual who was introduced as the co-actor. This, in turn, may make it more likely for participants to also ascribe additional attributes to them that are usually associated with their status, like low competence, or to trigger spontaneous tendencies of avoidance or disgust that are sometimes individuals with low status (Durante et al., 2017). This could affect the degree to which participants co-represent the co-actor’s actions.

A similar argument can be made for the low status condition. Certain factors like a positive relationship (Hommel et al., 2009) have been shown to increase the size of the joint Simon effect and also the size of oSRBR effects can differ depending on specific manipulations used to induce interdependence (Giesen et al., 2014; Iani et al., 2011). Therefore, even if the task or the status manipulation themselves induced enough interdependence between participants for oSRBR effects to occur, if a higher status partner really had been regarded as much more relevant than a lower status partner and therefore attracted more attention, this should have resulted in bigger effects in the low status compared to the high status condition. Since we did not find this, this implies that the power manipulation did not render the interaction partner particularly worthy of imitation in the eyes of the participants in the given, already collaborative, context. This may again be due to the fact that power was just a role that was assigned randomly in our experiment, therefore possibly preventing inferences of leader-related positive personal attributes typically associated with high status (Durante et al., 2017; Mattan et al., 2017).

From the arguments made so far, two suggestions for future research can be derived: Firstly, it should be investigated whether social status can modulate binding and retrieval processes when the context itself does not already induce interdependence between interaction partners. Second, it would be interesting to test whether the influence on oSRBR effects is stronger for social status manipulations that appear to be based on (ostensibly) authentic personal information about the partner, which should make it more likely for participants to make inferences about whether or not they are worthy of imitation.

Note that the possibility that social status manipulated by power does not influence oSRBR effects at all also needs to be considered. Although there are several studies that found a modulation of joint action or imitation effects by social status manipulations (Aquino et al., 2015; Farwaha & Obhi, 2021a, b; Tufft, 2022; van der Weiden et al., 2021), this was not the case in all studies (Farmer et al., 2016; see Supplement Table 5 for an overview over all studies and their status manipulations). Thus, the evidence on the influence of social status on imitative or joint action regulation is somewhat mixed. At the moment, it is difficult to account for these mixed findings, as the manipulation of status/power were rather diverse and very different across the studies: That is either because it is unclear whether the observed modulation was truly due to status/power differences (van der Weiden et al., 2021) or because these studies used manipulations that differ significantly from ours (Aquino et al., 2015; Farwaha & Obhi, 2021a, 2021b; Tufft, 2022) and therefore investigated different aspects of social status. As mentioned above, both Aquino et al. (2015) and Tufft (2022) used (seemingly) authentic information about the interaction partner to manipulate their perceived status. Such a manipulation likely does not only influence how powerful the interaction partner is perceived to be, but other status-related attributes like competence may also be affected. The studies by Farwaha and Obhi (2021a, b), on the other hand, did not manipulate the perceived status of an interaction partner, but how powerful or prestigious participants themselves felt. Thus, although results from previous studies suggest that some forms of social status can modulate joint action and imitation effects, it remains unclear whether this also holds true for the perceived power of one’s interaction partner.

### Limitations

One limitation of the present study is that we cannot fully exclude the possibility that conducting the study in an online rather than in a face-to-face setting may have affected the results. So far, all experiments that found a modulation of oSRBR effects by social relevance in interactions between two humans were conducted in the lab and did not use a social status manipulation (Giesen et al., 2014, 2018). The fact that the partner was not physically present might have led participants to perceive the situation as less interactive and less individualized, rendering it less sensitive to the influence of the other’s actions and of the social relevance manipulation in general. An anonymous online setting may be suboptimal for inferring and ascribing attributes to the partner, or for eliciting strong emotional-motivational responses. Future research could aim at overcoming this limitation. As explained above, there are studies that found a modulation of joint action effects by social status (Aquino et al., 2015), in one case in an online context (Tufft, 2022). These studies used manipulations based on (seemingly) real information about the co-actors. Therefore, introducing personalized information (pictures of a person or their belongings, or videos of another person) into the online setting might make status related inferences and responses more likely, and more targeted. A specific individual may be necessary to whom various attributes and can be attached and to whom motivational/emotional responses can be directed. In an anonymous situation, however, there is no object of disgust/admiration etc. which might undermine the potency of the manipulation and its implications for oSRBR effects.

Another concern that may arise for an interactive online study is whether participants actually believed that their interaction partner was indeed another human. This is important since both binding and retrieval by observation (Giesen & Rothermund, 2022) and other imitative effects like automatic imitation (Gowen et al., 2016; Klapper et al., 2014; Liepelt & Brass, 2010; Liepelt et al., 2010; Press et al., 2006; Stanley et al., 2007) and the joint Simon effect (Müller et al., 2011; Tsai & Brass, 2007; Tsai et al., 2008) are usually reduced or even absent if people do not believe that their partner is an animate, biological or human being. However, we consider this unlikely to be an issue here, as for both experiments most participants indicated that while doing the task they had believed that their partner was human. Also, excluding the participants that expressed doubts about their partner’s identity did not affect results (see supplementary material).

Finally, our results may be specific to the cultural background of our sample. While social hierarchies exist in every culture (Cheng & Tracy, 2014), some cultures are more hierarchical than others (Hofstede, 1984, 2001). Therefore, the susceptibility for a status manipulation based on power and the impact such a manipulation has on one’s cognition may also differ based on the cultural background of the specific sample. We only recruited participants from Germany and the UK, which are considered less hierarchical (Hofstede, 2001). Thus, it is possible that the power manipulation was not significant enough for our sample to affect oSRBR effects. This might be different in more hierarchical cultures, which is an avenue for future research.

### Conclusions

Social status manipulations – though effective in shaping perceptions of high versus low status – did not modulate oSRBR effects in two experiments. The low status condition either did not render the partner irrelevant enough and/or the high status condition did not render the interaction partner as even more relevant than they already were for differences in oSRBR effects to show. However, given the diversity of ways in which social status can be experimentally induced, it would be premature to claim that no form of social status could affect oSRBR effects. For future research, it would be interesting to investigate whether social status manipulations based on something other than power yield different results. Specifically, these manipulations should appear to reflect authentic information about the interaction partner, making it more likely for participants to make inferences about whether they are worthy of imitation in the context of the task.

## Supplementary Information

Below is the link to the electronic supplementary material.

Supplementary Material 1 (PDF 403 KB)

## Data Availability

The data is available at the following OSF repository: https://osf.io/d6wq2/6https://osf.io/d6wq2/6

## Footnotes

1 Although the status manipulation was successful overall, there were a few participants in both experiments whose status score did not correspond to their assigned status group, indicating that our manipulation did not work for these participants. Excluding these participants from the main analysis did not change our results (see supplementary material for details), i.e., there was still no modulation of retrieval of observationally acquired SR bindings by social status. Furthermore, correlations between status scores and oSRBR effects were also not significant (Exp. 1: *r* =.08, *p* =.31; Exp. 2, *r* = −.07, *p* =.38).

2 The inclusion of the memory test also may have led to participants generally responding slower in Experiment 1 compared to Experiment 2. Assuming that the retrieval of observationally acquired SR bindings from memory takes some time, this would suggest that there was less room for oSRBR effects to occur in Experiment 2 because of faster response times overall. If this were true, then oSRBR effects should be present for trials with slower RT. We addressed this question with an exploratory analysis that added RT bin(quartiles; cf. Ratcliff, 1979; Hommel, 1997) as additional factor. Notably, this analysis revealed no such modulation, as oSRBR effects were absent even for comparably slow RT bins in Experiment 2 (see supplementary material), excluding this alternative explanation.
