# Supplementary material for: You have no power here! Social status does not modulate observationally acquired binding and retrieval effects
Source: Psychol Res. 2025 Sep 30;89(5):148. doi: 10.1007/s00426-025-02191-4 (PMC12484378; doi:10.1007/s00426-025-02191-4)
Supplement: Supplementary file 1 — Supplementary Material 1 (PDF 403 KB) [file 426_2025_2191_MOESM1_ESM.pdf]

Supplementary material

To ascertain that the present null findings, i.e. the absence of the expected three-way interaction, is not due other causes, we ran exploratory analyses. A first analysis only regarded participants whose felt power rating score corresponded with the assigned status condition, as the manipulation might not have affected all participants to the same degree. A second exploratory analysis across experimental halves explores whether the expected modulatory influences of social status on oSRBR effects is a fleeting phenomenon that quickly disappears over time (cf. Giesen et al., 2021). A third exploratory analysis investigates whether participants might not have believed to be interacting with another human partner, which would explain smaller or absent retrieval effects. A forth exploratory analysis tests whether oSRBR effects and modulatory influences of social status were stronger for slower RT bins. Fifth, we ran a joint analysis of both experiments to further support the null finding and also contrast both experiments in a mini-metaanalysis. Finally, we provide a table summarizing status manipulations and results of previous studies investigating social status or power manipulations in related paradigms.

Analysis with participants whose status score corresponded to their status condition only

As preregistered, we also checked whether excluding participants whose status score did not correspond to their assigned status condition, i.e. negative scores in the high status condition (Experiment 1: n = 15; Experiment 2: n = 18) and positive scores in the low status condition (Experiment 1: n = 16; Experiment 2: n = 17). After exclusion, we analyzed probe performance of the remaining sample (Experiment 1: n =135, high status: n = 67, low status: n = 68; Experiment 2: n = 128, high status: n = 64, low status: n = 64), using a 2 (stimulus relation: stimulus repetition vs. stimulus change) x 2 (response compatibility: compatible vs. incompatible) x 2 (status: high vs. low) mixed factors ANOVA (see Supplement Table 1 for means). For Experiment 1 the ANOVA revealed a significant main effect of stimulus relation, $F(1, 133) = 10.1$, $p = .002$, $\eta^2_p = .07$, showing that participants responded faster when words

Supplement Table 1

Probe performance M (SD) in the observational SR binding paradigm, only participants whose status score corresponded to their status group

		High status		Low status	
		C	IC	C	IC
Experiment 1	Stimulus repetition (SR)	512 (75)	511 (70)	491 (71)	497 (70)
	Stimulus change (SC)	520 (77)	508 (71)	500 (74)	497 (68)
	Δ SC - SR	8** [3.2]	-3 [2.1]	9*** [2.4]	0 [2.5]
	S x R interaction score	12** [3.7]		9* [3.9]	
Experiment 2	Stimulus repetition (SR)	490 (68)	485 (69)	479 (57)	474 (61)
	Stimulus change (SC)	492 (69)	489 (74)	482 (60)	474 (60)
	Δ SC - SR	2 [2.2]	4 [2.2]	3 [2.0]	0 [2.5]
	S x R interaction score	-2 [3.2]		3 [2.8]	

Note. C =compatible probe response, IC= incompatible probe response. S x R interaction score = $(\Delta \text{ SC - SR})_{C}$ - $(\Delta \text{ SC - SR})_{IC}$. Standard error of the mean in brackets. * $p < .05$. ** $p < .01$. *** $p < .001$. Asterisks denote that effects significantly differ from zero

repeated from prime to probe (M = 502 ms) than when they changed (M = 506 ms). Additionally, there was a significant interaction of stimulus relation and response compatibility, $F(1, 133) = 14.3$, $p < .001$, $\eta^2_p = .10$, implying the presence of oSRBR effects. However, these were not modulated by social status, as indicated by the absence of a significant three-way interaction, $F(1, 133) = 0.27$, $p = .604$, $\eta^2_p < .01$. All other effects were not significant either, all $F \leq 3.42$, all $p \geq .067$.

For Experiment 2 the analysis revealed significant main effects of stimulus relation, $F(1, 126) = 4.45$, $p = .037$, $\eta^2_p = .03$, with faster responses for repetitions (M = 482 ms) vs. changes (M = 484 ms), and response compatibility, $F(1, 126) = 12.5$, $p = .001$, $\eta^2_p = .09$, indicating faster responses when prime and probe responses were incompatible (M = 481 ms) than when responses were compatible (M = 486 ms). All other effects, including the stimulus relation and response compatibility interaction and the three-way interaction, were

not significant, all $F \leq 1.06$, all $p \geq .304$, implying that there were no oSRBR effects and no modulation of those by social status.

Supplement Table 2

Probe performance M (SD) in the observational SR binding paradigm by blocks

		High status		Low status	
		C	IC	C	IC
Experiment 1		Block 1			
	Stimulus repetition (SR)	513 (76)	510 (70)	501 (81)	502 (77)
	Stimulus change (SC)	527 (78)	513 (73)	514 (85)	504 (74)
	Δ SC - SR	14** [3.9]	3 [3.2]	13*** [2.9]	2 [3.0]
	S x R interaction score	11* [4.8]		11* [4.4]	
		Block 2			
	Stimulus repetition (SR)	499 (76)	502 (75)	495 (77)	504 (78)
	Stimulus change (SC)	502 (77)	494 (73)	499 (81)	496 (72)
	Δ SC - SR	3 [3.5]	-8* [2.9]	4 [3.9]	-8* [3.5]
	S x R interaction score	11* [4.8]		12* [5.2]	
Experiment 2		Block 1			
	Stimulus repetition (SR)	489 (69)	485 (70)	472 (57)	468 (55)
	Stimulus change (SC)	494 (70)	486 (75)	477 (61)	467 (58)
	Δ SC - SR	5 [2.8]	1 [2.9]	5 [2.9]	-1 [2.9]
	S x R interaction score	4 [3.9]		6 [4.1]	
		Block 2			
	Stimulus repetition (SR)	493 (74)	485 (75)	481 (57)	475 (63)
	Stimulus change (SC)	492 (73)	490 (77)	483 (60)	477 (58)
	Δ SC - SR	-1 [2.9]	5 [3.4]	2 [2.2]	2 [2.4]
	S x R interaction score	-6 [4.4]		0 [3.2]	

Note. C = compatible probe response, IC= incompatible probe response. S x R interaction score = $(\Delta \text{SC - SR})_{C}$ - $(\Delta \text{SC - SR})_{IC}$. Standard error of the mean in brackets. * $p < .05$. ** $p < .01$. *** $p < .001$. Asterisks denote that effects significantly differ from zero

Block analysis

To test whether oSRBR effects changed over time, we added "block" as an additional within-factor to the factorial design. Block had two levels: "1" including all trials of the first half of the observational SR binding task, and "2" included all trials of the second half. Then, we computed mean probe RTs for all conditions of the factorial design including this new factor (see Supplement Table 2). Mean RTs were entered into a 2 (stimulus relation: stimulus repetition vs. stimulus change) x 2 (response compatibility: compatible vs. incompatible) x 2 (status: high vs. low) x 2 (block: 1 vs. 2) mixed factors ANOVA. For Experiment 1 the ANOVA revealed significant main effects of stimulus relation, $F(1, 164) = 7.61$, $p = .006$, $\eta^2_p = 0.04$,

Figure S1

Probe performance (RT) in Experiment 2 as a function of stimulus relation, response compatibility, status condition and block

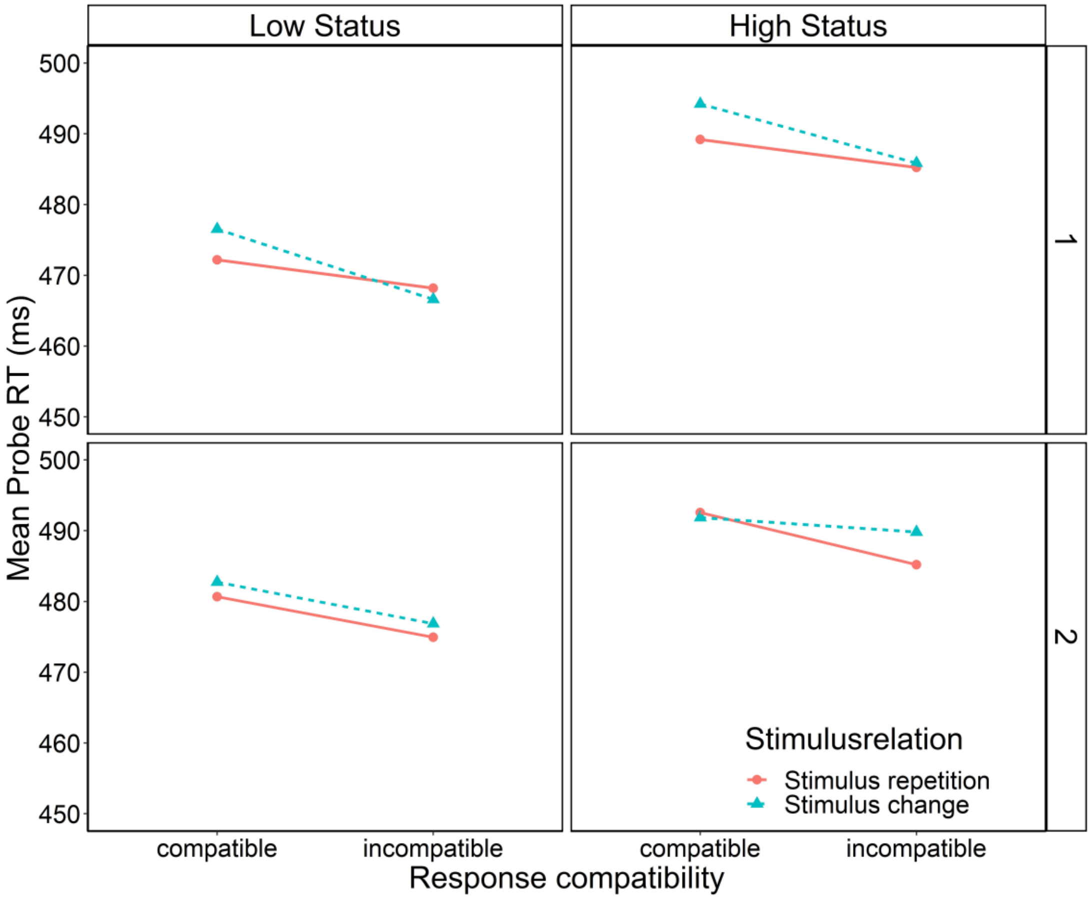

Note. "1" and "2" refer to the first and the second block respectively

indicating that participants responded faster when stimuli repeated from prime to probe (M = 503 ms) than when stimuli changed (M = 506 ms), and block, $F(1, 164) = 26.5$, $p < .001$, $\eta^2_p = 0.14$, with faster responses in the second (M = 499 ms) vs. the first block (M = 510 ms). Further, there were significant interactions of stimulus relation and response compatibility, $F(1, 164) = 21.8$, $p < .001$, $\eta^2_p = 0.12$, status and block, $F(1, 164) = 5.30$, $p = .023$, $\eta^2_p = 0.03$, word relation and block, $F(1, 164) = 16.5$, $p < .001$, $\eta^2_p = 0.09$, and response compatibility and block, $F(1, 164) = 7.26$, $p = .008$, $\eta^2_p = 0.04$. All other effects were not significant (all $F \leq 3.11$, all $p \geq .080$), including the three-way interaction of stimulus relation, response compatibility and block, $F(1, 164) = 0.00$, $p = .945$, $\eta^2_p < 0.01$, and the four-way interaction, $F(1, 164) = 0.01$, $p = .928$, $\eta^2_p < 0.01$, indicating that neither retrieval effects nor their modulation by status changed over time in Experiment 1.

Results for Experiment 2 revealed a significant main effect of response compatibility, $F(1, 161) = 21.9$, $p < .001$, $\eta^2_p = .12$, with participants responding faster if responses changed from prime to probe (M = 479 ms) than when they repeated (M = 485 ms). There was also a main effect of block, $F(1, 161) = 4.81$, $p = .030$, $\eta^2_p = .03$, showing that responses on average were faster in the first half of the experiment (M = 480 ms) compared to the second half (M = 484 ms). All other effects were not significant. However, the three-way interaction of stimulus relation, response compatibility and block almost reached significance, $F(1, 161) = 3.75$, p = .055, $\eta^2_p = .02$, indicating that the size of the retrieval effect (represented by the stimulus relation x response compatibility interaction) differed between the first and the second half of Experiment 2 (see Figure S1).

Analysis with participants who believed their partner was human only

The belief that the partner is not human has been found to eliminate oSRBR effects (Giesen & Rothermund, 2022). Since some of our participants also doubted that their partner was another person, we were interested in whether this affected our results. First, we excluded all participants (Experiment 1: n = 30; Experiment 2: n = 50), who had indicated that already while doing the experiment they did not believe they were interacting with

Supplement Table 3

Probe performance M (SD) in the observational SR binding paradigm, only participants who believed their partner was human

		High status		Low status	
		C	IC	C	IC
Experiment 1	Stimulus repetition (SR)	500 (70)	502 (66)	499 (77)	504 (76)
	Stimulus change (SC)	511 (73)	500 (67)	508 (81)	502 (73)
	Δ SC – SR	11** [3.0]	-2 [2.1]	9** [2.5]	-2 [2.9]
	S x R interaction score	13** [3.6]		11** [3.9]	
Experiment 2	Stimulus repetition (SR)	489 (74)	482 (75)	476 (58)	470 (58)
	Stimulus change (SC)	492 (77)	485 (81)	480 (59)	471 (60)
	Δ SC – SR	3 [2.3]	3 [2.4]	4* [1.8]	1 [2.2]
	S x R interaction score	0 [3.5]		3 [2.5]	

Note. C = compatible probe response, IC= incompatible probe response. S x R interaction score = $(\Delta \text{ SC - SR})_{C}$ - $(\Delta \text{ SC - SR})_{IC}$. Standard error of the mean in brackets. * *p* < .05. ** *p* < .01. *** *p* < .001. Asterisks denote that effects significantly differ from zero

another human. With the remaining sample (Experiment 1: *n* = 136, high status: *n* = 71, low status: *n* = 65; Experiment 2: *n* = 113, high status: *n* = 56, low status: *n* = 57), we conducted a 2 (stimulus relation: stimulus repetition vs. stimulus change) x 2 (response compatibility: compatible vs. incompatible) x 2 (status: high vs. low) mixed factors ANOVA on mean probe RTs (see Supplement Table 3 for means). For Experiment 1, the ANOVA revealed a significant main effect of stimulus relation $F(1, 134) = 8.03$, $p = .005$, $\eta^2_p = .06$, with faster responses for stimulus repetitions (*M* = 501 ms) compared to stimulus changes (*M* = 505 ms), and a significant interaction of stimulus relation and response compatibility, $F(1, 134) = 18.8$, $p < .001$, $\eta^2_p = .12$, indicating retrieval of observationally acquired SR bindings. Consistent with the results of the main ANOVA that included all participants, however, the three-way interaction was not significant, $F(1, 134) = 0.03$, $p = .857$, $\eta^2_p < .01$, meaning that retrieval effects were not modulated by social status. All other effects were not significant, all $F \leq 1.68$, all $p \geq .197$.

For Experiment 2, there were only significant main effects of stimulus relation, $F(1, 111) = 4.40$, $p = .038$, $\eta^2_p = .04$, with faster responses for stimulus repetitions ($M = 479$ ms) compared to stimulus changes ($M = 482$ ms), and response compatibility, $F(1, 111) = 23.2$, $p < .001$, $\eta^2_p = .17$, indicating that participants on average responded faster when responses changed ($M = 477$ ms) from prime to probe than when responses repeated ($M = 484$ ms). All other effects, including the two-way interaction of stimulus relation and response compatibility and the three-way interaction, were not significant, all $F \leq 1.02$, all $p \geq .314$. This implies that there was no retrieval of observationally acquired SR bindings overall and no modulation by social status.

Testing for Modulations by RT quartiles

In Experiment 2, response times were generally faster than in Experiment 1 (Experiment 1: $M = 505$ms, Experiment 2: $M = 482$ms). We can only speculate why this was the case. As one possibility, the presence of the memory test might have slowed participants performance in the task overall. Assuming that it takes some time for observationally acquired SR bindings to be retrieved from memory, an alternative explanation for the absence of oSRBR effects in Experiment 2 could be that the faster responses left less room for retrieval to occur. If this is indeed the case, then oSRBR effects should be stronger for slower responses. This, in turn, would mean that modulations by social status might be easier to detect for slower RT bins. To test this, RTs were vincentized according to the procedure suggested by Ratcliff (1979). That means, for each participant individual mean RT quartiles were calculated of the rank-ordered raw data for each combination of stimulus relation and response compatibility. Then we computed mean probe RTs for each condition of the factorial design for each quartile (see Supplement Table 4 for means). Mean RTs were entered into a 2 (stimulus relation: stimulus repetition vs. stimulus change) x 2 (response compatibility: compatible vs. incompatible) x 2 (status: high vs. low) x 4 (RT quartile: 1 vs. 2 vs. 3 vs. 4) mixed factors ANOVA. As long as the quartile factor is not involved in an interaction, all statistical effects of this analysis are identical to that of a standard ANOVA of conventionally computed means (i.e. without the quartile factor) and were already reported

Supplement Table 4

Probe performance M (SD) in the observational SR binding paradigm by quartiles

		High status		Low status	
		C	IC	C	IC
Experiment 1		1st Quartile			
	Stimulus repetition (SR)	419 (54)	420 (52)	411 (58)	414 (56)
	Stimulus change (SC)	428 (61)	423 (54)	417 (62)	417 (58)
	Δ SC - SR	9*** [2.3]	3 [2.1]	6** [2.1]	3 [2.4]
	S x R interaction score	6* [2.7]		3 [3.0]	
		2nd Quartile			
	Stimulus repetition (SR)	472 (67)	473 (64)	464 (72)	469 (67)
	Stimulus change (SC)	483 (70)	472 (63)	472 (74)	468 (66)
	Δ SC - SR	9** [3.1]	-1 [2.1]	8** [2.4]	-1 [2.7]
	S x R interaction score	10** [3.8]		9** [3.7]	
		3rd Quartile			
	Stimulus repetition (SR)	523 (80)	523 (76)	514 (82)	522 (81)
	Stimulus change (SC)	533 (79)	517 (75)	526 (86)	516 (77)
	Δ SC - SR	10* [3.6]	-6* [2.6]	12*** [3.0]	-6 [3.1]
	S x R interaction score	16** [4.8]		18*** [4.6]	
		4th Quartile			
	Stimulus repetition (SR)	609 (98)	606 (94)	603 (105)	605 (102)
	Stimulus change (SC)	615 (98)	602 (98)	611 (105)	599 (93)
	Δ SC - SR	6 [4.0]	-4 [3.6]	8* [3.6]	-6 [3.8]
	S x R interaction score	10 [5.3]		14** [5.2]	
Experiment 2		1st Quartile			
	Stimulus repetition (SR)	410 (51)	407 (52)	405 (41)	401 (42)
	Stimulus change (SC)	413 (51)	409 (54)	409 (44)	400 (43)
	Δ SC - SR	3 [2.0]	2 [2.2]	4** [1.7]	-1 [2.0]
	S x R interaction score	1 [2.7]		5* [2.5]	
		2nd Quartile			
	Stimulus repetition (SR)	463 (65)	458 (64)	451 (50)	446 (53)
	Stimulus change (SC)	464 (65)	460 (67)	455 (53)	444 (52)
	Δ SC - SR	1 [2.4]	2 [1.9]	4* [1.9]	-2 [2.2]
	S x R interaction score	-1 [3.0]		6* [3.1]	

Supplement Table 4 Continued

	High status		Low status	
	C	IC	C	IC
	3rd Quartile			
Stimulus repetition (SR)	508 (74)	501 (76)	492 (60)	486 (63)
Stimulus change (SC)	510 (76)	505 (80)	493 (61)	486 (60)
Δ SC - SR	2 [2.5]	4 [2.5]	1 [2.1]	0 [2.8]
S x R interaction score	-2 [3.8]		1 [3.4]	
	4th Quartile			
Stimulus repetition (SR)	583 (103)	576 (103)	558 (74)	552 (76)
Stimulus change (SC)	584 (97)	577 (102)	560 (78)	555 (76)
Δ SC - SR	1 [3.8]	1 [3.7]	2 [3.2]	3 [3.4]
S x R interaction score	0 [4.9]		-1 [3.8]	

Note. C = compatible probe response, IC= incompatible probe response. S x R interaction score = $(\Delta\ SC - SR)_{C} - (\Delta\ SC - SR)_{IC}$. Standard error of the mean in brackets. * $p < .05$. ** $p < .01$. *** $p < .001$. Asterisks denote that effects significantly differ from zero

in the results section. Therefore, in the following we will only report effects involving the quartile factor.

For Experiment 1, there was a significant main effect of quartile ($F(3, 492) = 1773.8$, $p < .001$, $\eta^2_p = .92$) and significant two-way interactions of quartile with both stimulus relation ($F(3, 492) = 3.39$, $p = .037$, $\eta^2_p = .02$) and response compatibility (($F(3, 492) = 4.73$, $p = .011$, $\eta^2_p = .03$). The ANOVA also revealed a significant three-way interaction of stimulus relation, response compatibility and quartile ($F(3, 492) = 5.27$, $p = .005$, $\eta^2_p = .03$). Importantly, however, the four-way interaction was not significant ($F(3, 492) = 0.82$, $p = .441$, $\eta^2_p = .01$). All other effects were not significant (all $F \leq 0.82$, all $p \geq .441$). Taken together, this implies that although the size of the oSRBR effect differed between quartiles in Experiment 1, there is still no evidence for a modulation by social status (see Figure S2).

For Experiment 2, the analysis only revealed a main effect of quartile ($F(3, 483) = 1313.7$, $p < .001$, $\eta^2_p = .89$) and an interaction of status and quartile ($F(3, 483) =$

3.81, *p* = .049, η^2_p = .02). All other effects did not reach significance (all $F \leq 1.63$, all $p \geq$ .196), including the three-way interaction of stimulus relation, response compatibility and quartile ($F(3, 483) = 1.63$, *p* = .196, η^2_p = .01) and the four-way interaction ($F(3, 483) = 1.08$, *p* = .344, η^2_p = .01). This means that oSRBR effects did not differ significantly between slower compared to faster responses in Experiment 2, nor did the modulation of these by

Figure S2

S x R interaction scores for retrieval of observationally acquired SR bindings as a function of RT quartile and status condition (a) in Experiment 1 and (b) in Experiment 2

(a)

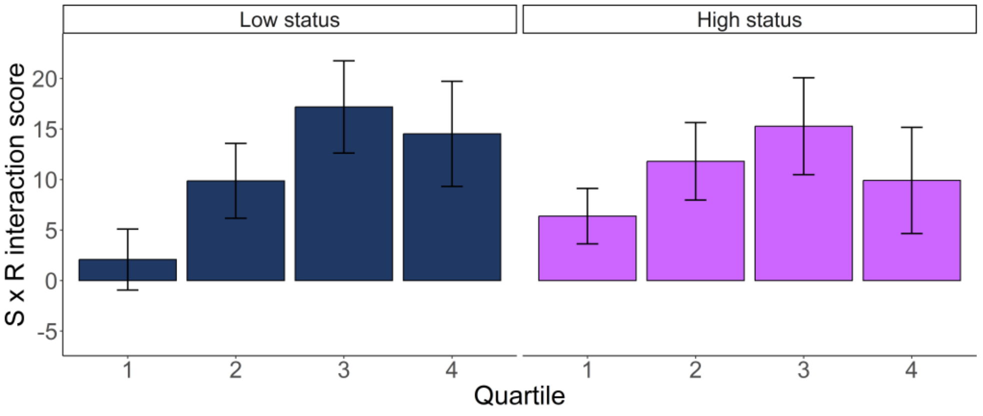

(b)

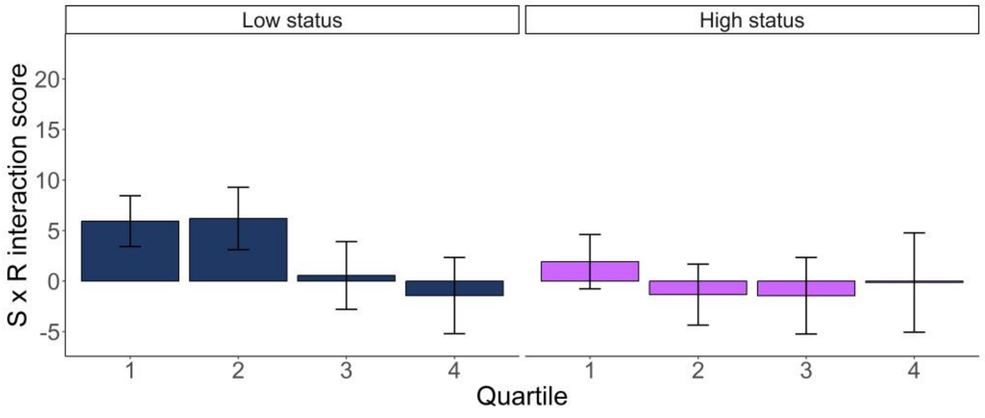

Note. S x R interaction scores are computed as net effects of stimulus repetition effects (Supplement Table 4) for compatible minus incompatible sequences (S x R = $(\Delta\ SC - SR)_{C} - (\Delta\ SC - SR)_{IC}$). Positive values reflect stimulus-based retrieval of observed responses. Error bars depict standard errors of the mean

social status. Descriptively, the data trend is in the other direction as the exploratory assumption – if anything, oSRBR effects tend to be present for faster bins, but absent for slower bins (see Figure S2). Together, this suggests that the absence of significant oSRBR effects cannot be explained by generally faster response times in Experiment 2.

Joint analysis

We combined the data of both experiments for a joint analysis to exclude the possibility that we did not find a modulation of oSRBR effects by social status due to insufficient power. A 2 (stimulus relation: stimulus repetition vs. stimulus change) x 2 (response compatibility: compatible vs. incompatible) x 2 (status: high status vs. low status) x 2 (experiment: 1 vs. 2) mixed factors ANOVA on mean probe RTs revealed significant main effects of stimulus relation ($F(1, 325) = 10.18$, $p = .002$, $\eta^2_p = .03$), with participants responding faster when words repeated from prime to probe ($M = 492$ ms) than when they changed ($M = 495$ ms), and of response compatibility ($F(1, 325) = 17.67$, $p < .001$, $\eta^2_p = .05$), indicating faster RTs for incompatible ($M = 496$ ms) vs. compatible ($M = 491$ ms) responses. There was also a main effect of experiment ($F(1, 325) = 9.15$, $p = .003$, $\eta^2_p = .03$), as responses on average were faster in Experiment 2 ($M = 482$ ms) than in Experiment 1 ($M = 505$ ms). Further, there was a significant two-way interaction of stimulus relation and response compatibility ($F(1, 325) = 16.47$, $p < .001$, $\eta^2_p = .05$), indicating the overall presence of oSRBR effects, and a three-way interaction of stimulus relation, response compatibility, and experiment ($F(1, 325) = 10.14$, $p = .002$, $\eta^2_p = .03$), meaning that the size of the oSRBR effect differed significantly between experiments. This is because significant oSRBR effects only emerged in Experiment 1, but not in Experiment 2 (see Results in main text). Most importantly, and consistent with the results from the separate analyses of both experiments, the three-way interaction of stimulus relation, response compatibility, and social status did not reach significance ($F(1, 325) = 0.27$, $p = .607$, $\eta^2_p < .01$), implying that retrieval of observationally acquired SR bindings did not differ between status groups. All other effects were not significant (all $F \leq 2.11$, all $p \geq .147$).

Additionally, we compared effects scores for retrieval of observationally acquired SR bindings using a one tailed, independent sample *t*-test for a direct test of our directional hypothesis. The *t*-test indicated that effect scores were not significantly larger in the low status condition than in the high status condition, $t(327) = 0.54$, $p = .296$, $d = 0.06$, $BF_{01} = 7.16$. *T*-tests against zero showed that effect scores differed significantly from zero in both the low, $t(164) = 3.30$, $p = .001$, $d = 0.26$, $BF_{01} = 0.06$; $M_{SxR} = 6.94$ ms, and the high status condition, $t(164) = 2.39$, $p = .018$, $d = 0.19$, $BF_{01} = 0.73$, $M_{SxR} = 5.29$ ms.

Mini-metaanalysis

In addition to the joint analysis, we performed a mini-metaanalysis of both experiments. This analysis yielded a mean effect size of $d = 0.06$, 95% CI (-0.15, 0.27) for modulatory effects of social status on retrieval of observationally acquired SR bindings (see Figure S3). As the confidence interval includes zero, this implies that the social status manipulation had no significant impact on oSRBR effects in our experiments.

Figure S3

Forest plot for mini-metaanalysis on modulatory effects of social status on oSRBR effects

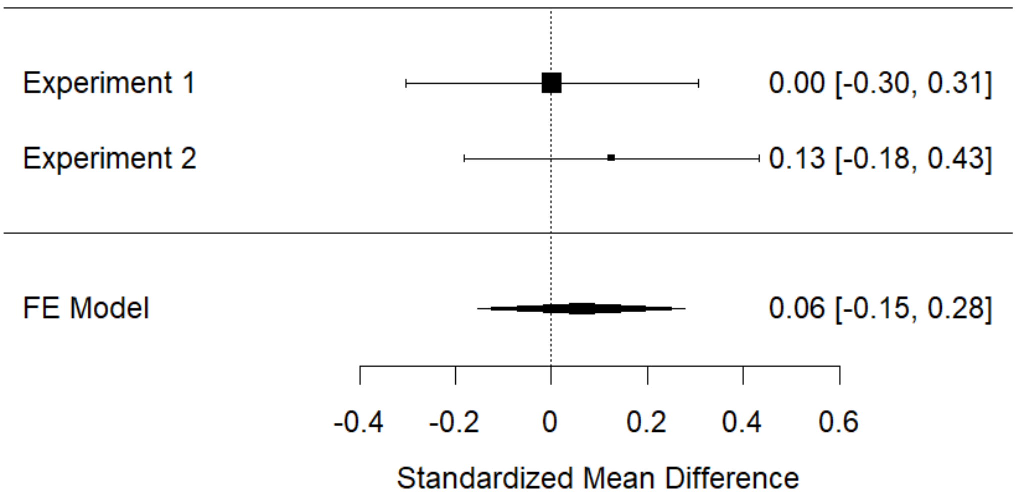

Supplement Table 5

Overview of previous studies investigating the influence of social status/power on joint action and imitation effects

Study	Investigated effect	Status manipulation	Central contrast	p-value / effect size
Aquino et al. (2015)	joint Simon effect	Co-actor's nationality: Albanian (lower status) vs. Italian (higher status)	Italian vs. Albanian co-actor	p = .012, N/A
Van der Weiden et al. (2021) – Experiment 1	joint Simon effect	Seating position: elevated (high-power) vs. lower (low-power)	Elevated vs. lower seating position	p < .001, N/A
Van der Weiden et al. (2021) - Experiment 2	joint Simon effect			p < .001, N/A
Farmer et al. (2016) – Experiment 1	automatic imitation	Associative learning task: models' faces either associated with high- or low-status characteristics	High vs. low status model	$p = .590$, $\eta^2 = .011$
Farmer et al. (2016) – Experiment 2	automatic imitation			$p = .847$, $\eta^2 = .002$
Farmer et al. (2016) – Experiment 3	automatic imitation	Perception of model's competence manipulated by better / worse performance in letter game compared to participant	High vs. low competence	$p = .982$, $\eta^2 = .002$
Farmer et al. (2016) – Experiment 4	automatic imitation	Assignment of power to make choices affecting a monetary payout	High vs. neutral vs. low power (of model)	$p = .14$, $\eta^2 = .108$
Farmer et al. (2016) – Experiment 5	automatic imitation			N/A (not significant), $\eta^2 < .001$
Farwaha and Obhi (2021a)	automatic imitation	Power priming by writing an essay about situation when participant had power over others / other had power over them	Low vs. high power priming	$p < .05$, $\eta_p^2 = .064$
Farwaha and Obhi (2021b) – Experiment 1	automatic imitation	Categorization of participants as Instagram "leader" vs. "follower" depending on the ratio of followers to followed profiles	Leaders vs. followers	$p = .003$, $\eta_p^2 = .200$
Farwaha and Obhi (2021b) – Experiment 2	automatic imitation			$p = .009$, $d = 0.479$
Tufft (2022)	social offloading	Visual cues (e.g. clothes, apartment) and CV information	High vs. low status	$p = .01$, $\eta_p^2 = .07$

Note. N/A indicates that the exact value was not provided in the paper
